# Mott transition in chain structure of strained VO_2_ films revealed by coherent phonons

**DOI:** 10.1038/s41598-017-16188-6

**Published:** 2017-11-22

**Authors:** Tetiana V. Slusar, Jin-Cheol Cho, Hyang-Rok Lee, Ji-Wan Kim, Seung Jo Yoo, Jean-Yves Bigot, Ki-Ju Yee, Hyun-Tak Kim

**Affiliations:** 1 Metal-Insulator-Transition Laboratory, Electronics and Telecommunications Research Institute, Daejeon, 34129 Republic of Korea; 2 Department of Advanced Device Technology, University of Science and Technology, Daejeon, 34129 Republic of Korea; 3 Department of Physics, Chungnam National University, Daejeon, 34134 South Korea; 4 Institut de Physique et Chimie des Matériaux de Strasbourg, UMR 7504, CNRS, Université de Strasbourg, 67034, Strasbourg, Cedex 02 France; 5 Center for Electron Microscopy Research, Korea Basic Science Institute, Daejeon, 34133 Republic of Korea

## Abstract

The characteristic of strongly correlated materials is the Mott transition between metal and insulator (MIT or IMT) in the same crystalline structure, indicating the presence of a gap formed by the Coulomb interaction between carriers. The physics of the transition needs to be revealed. Using VO_2_, as a model material, we observe the emergence of a metallic chain in the intermediate insulating monoclinic structure (M2 phase) of epitaxial strained films, proving the Mott transition involving the breakdown of the critical Coulomb interaction. It is revealed by measuring the temperature dynamics of coherent optical phonons with separated vibrational modes originated from two substructures in M2: one is the charge-density-wave, formed by electron-phonon (*e-ph*) interaction, and the other is the equally spaced insulator-chain with electron-electron (*e-e*) correlations.

## Introduction

Vanadium dioxide (VO_2_), a canonical transition metal oxide with strongly correlated electrons, undergoes the insulator-to-metal transition (IMT) near *T*_*IMT*_ = 340 K, accompanied by the structural phase transition (SPT) between the monoclinic and rutile (R) phases. The IMT mechanism is still under debate, because the IMT occurs near the SPT and the complicated structure shields the driving mechanism of the phase transformation^1–4^. It has been argued whether the IMT is the Mott transition^5–10^ driven by the breakdown of electron-electron (*e-e*) correlations, the Peierls transition^11–13^ induced by melting of the charge density wave (CDW) formed by electron-phonon (*e-ph*) interactions, or the Mott-Peierls transition^14,15^ occurring by means of both effects (*e-e* and *e-ph*). The microscopic understanding of the *e-e* and *e-ph* interactions, regarded as the IMT in doped semiconductors^4,16^, Mott insulators^17,18^, high temperature superconductors^19^, layered transition metal dichalcogenides^20^, is important for science and technology.

The VO_2_ below *T*_*IMT*_ has the monoclinic structure (M1) and the second monoclinic structure (M2) with two kinds of substructures of V-atoms, which are a CDW substructure with lattice distortion and an antiferromagnetic equally spaced insulator-chain (IC) one. The M2 phase, as an intermediate between M1 and R, was discovered in Cr-doped^21,22^ and strained^23–26^ VO_2_ samples. It enables the Mott IMT scenario implying transformation from the IC to a metallic chain (MC), which occurs by excitation of charges in the indirect impurity band formed by such as oxygen vacancies leading to the chain collapse of the main band^27^.

Numerous works have been published with both theoretical and experimental evidence for the metallization of the monoclinic phase and, thus, for the Mott transition^5–10,27–33^. Among them, Tao, *et al*.^30^, monitored VO_2_ microbeams on heating using optical, transmission electron microscopy, and ultrafast electron diffraction techniques and showed that charge doping stabilizes a new monoclinic metal phase prior to the SPT. Morrison, *et al*.^31^, used an ultrafast electron diffraction technique with infrared transmissivity to separate the optically-induced charge and lattice reorganizations in polycrystalline VO_2_ films. Wegkamp, *et al*.^32^, performed femtosecond time-resolved photoelectron spectroscopy of photoexcitated thin VO_2_ films and obtained an instantaneous pure electronic transition excluding any structural bottleneck. J. Laverock, *et al*.^33^, observed a temperature-triggered Mott transition in VO_2_ with the help of low-energy electron microscopy and photoemission spectroscopy. Li, *et al*.^15^, measured the monoclinic metallic phase under high pressure.

Despite intensive studies of the IMT, the two substructures in the monoclinic metallic phase (MMP), CDW and MC, have not been separately observed for the Mott transition. Moreover, the MC has never been experimentally proven. Thus, there has been no decisive argument for the Mott transition.

Here, we report a first observation of the metallic chain (MC) in the monoclinic structure of strained VO_2_ epifilms grown on an AlN/Si substrate^34^, providing strong evidence of the Mott transition. This is achieved by analyzing coherent phonons, measured below a photo-induced (thermal and non-thermal component) IMT threshold^35,36^ by ultrafast pump-probe spectroscopy. This is a unique tool capable of simultaneously sensing both structural reconstruction and electronic system transformation on a femtosecond timescale^37–41^. Moreover, our results show that the dynamics of the phonons on heating reveal not only the structural reconstruction of VO_2_ during transitions of the M1 insulator → M2 insulator → Rutile metal, but also the intrastructural rearrangement of the V-chains in the M2 phase.

## Results and Discussions

**Figure 1:**
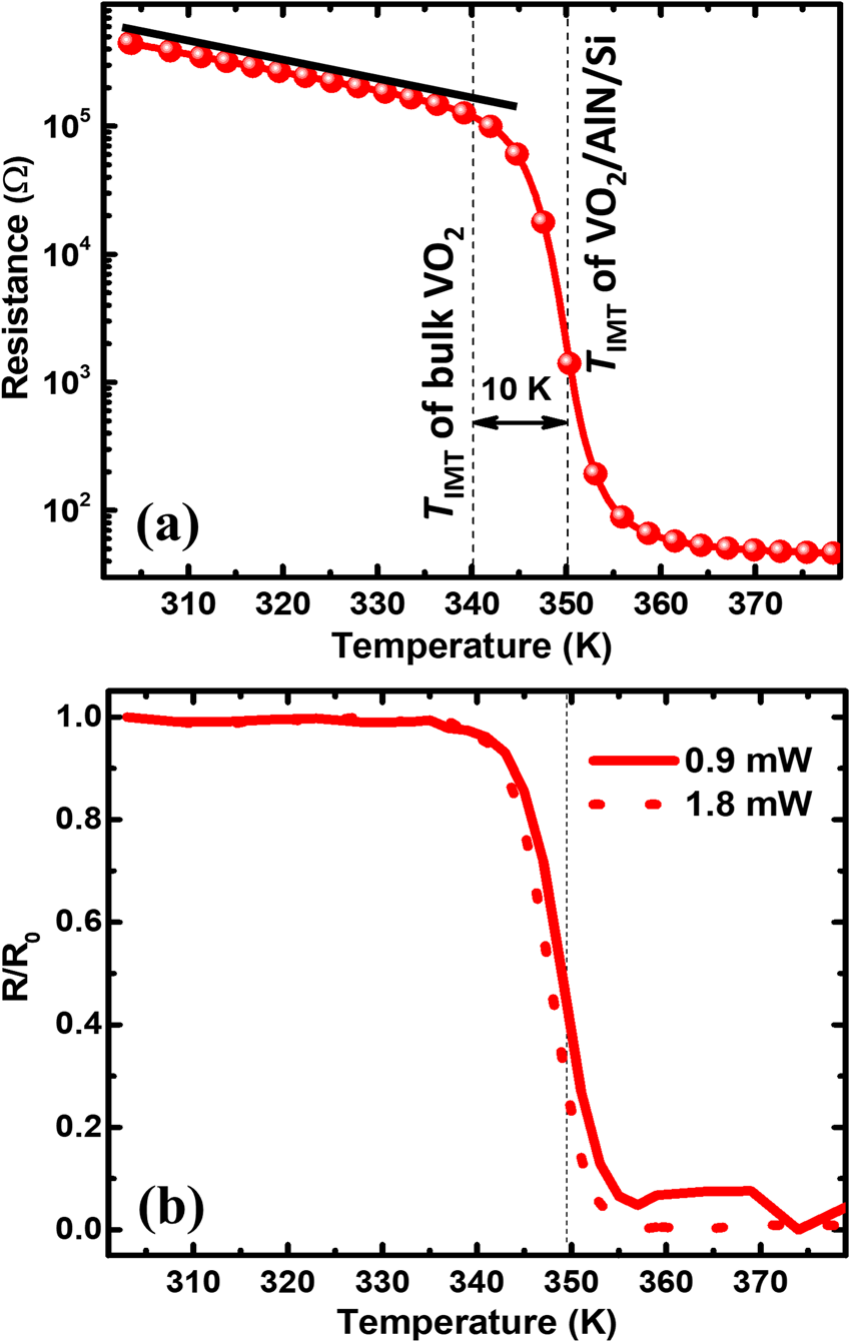
(**a**) Temperature dependence of resistance of the strained 120 nm thick VO_2_ film on an AlN/Si substrate. An insulator-to-metal transition temperature (*T*_IMT_) is approximately 350 K, which is 10 K higher than *T*_IMT_ of the bulk sample. (**b**) Normalized probe reflectivity (*R/R*_0_) at pump powers of 0.9 mW (2.1 mJ/cm^2^) and 1.8 mW (4.2 mJ/cm^2^) measured at different temperatures of VO_2_/AlN/Si.

Figure 1a shows the temperature dependence of electrical resistance for 120 nm thick VO_2_ film on AlN/Si with a critical transition temperature *T*_IMT_ ≈ 350 K higher than the typical value of bulk or bulk-like VO_2_ samples with *T*_IMT_ ≈ 340 K. This is caused by the substrate-induced strain in the film, as demonstrated by high resolution transmission electron microscopy (see Fig. S4 in the Supplementary Information), which stabilizes the insulating phase and, therefore, drives the 10 K-upshift of *T*_IMT_^34^. As shown below, this peculiarity of the sample enables the extended existence of the monoclinic crystalline structure and its electronic insulator-to-metal switch at 350 K.

**Figure 2:**
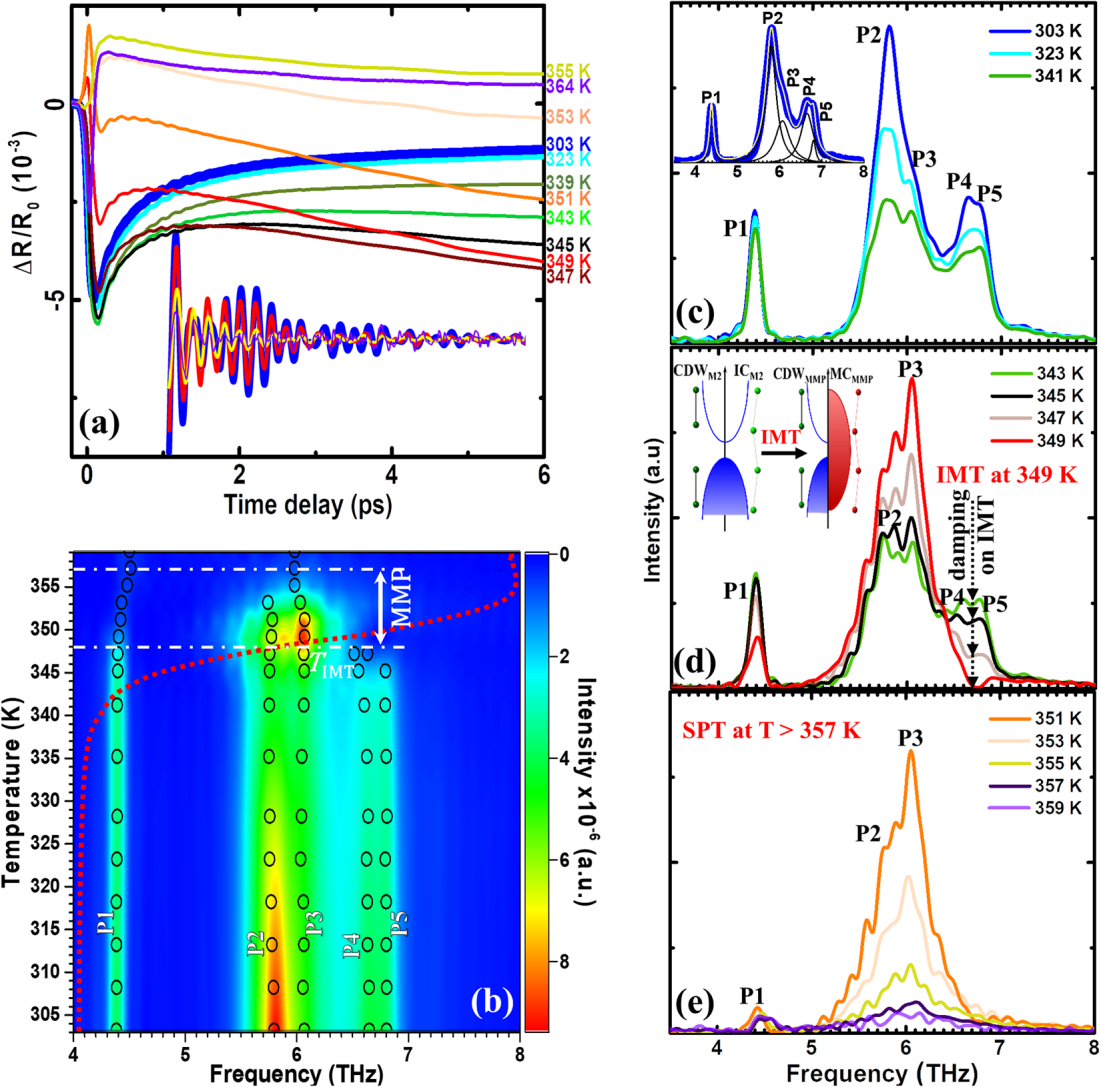
(**a**) Temperature dependence of transient reflectivity with the extracted component of coherent phonons (inset) for the VO_2_/AlN/Si sample. (**b**) Temperature-frequency map of the five coherent phonon modes (P1-P5, black circles) with *R*/*R*_0_ red-dotted curve from Fig. 1b, simultaneously revealing the structural and electronic states of VO_2_ on AlN/Si. (**c**–**e**) Representative FFT spectra obtained at different temperatures. Inset of Fig. 2c shows an example of the Lorenzian function fitting of the FFT spectrum taken at 303 K. Inset of Fig. 2d shows the band diagrams for the semiconducting (or insulating) VO_2_ with the CDW_M2_ and IC_M2_ substructures (before IMT) and for the semiconducting (or insulating) CDW_MMP_ and metallic MC_MMP_ substructures (after IMT).

Figure 1b shows the temperature dependence of normalized probe reflectivity (*R/R*_0_) measured at two different pump powers, 0.9 and 1.8 mW, corresponding to the fluences of 2.1 and 4.2 mJ/cm^2^, respectively. Both *R/R*_0_ curves, in accordance with the resistance switching (Fig. 1a), exhibit the same drastic changes at about 350 K without any fluence dependence. Thus, the pump laser with a fluence up to 4.2 mJ/cm^2^ is low enough not to affect phase transition of the sample, in contrast to higher fluences as observed in^42,43^. Further optical measurements, as presented in Fig. 2, were performed at a pump fluence of 2 mJ/cm^2^.

Figure 2a shows the temperature dependence of transient reflectivity Δ*R*/*R*_0_ measured for VO_2_/AlN/Si. Here, at low temperatures of 303–323 K (blue and cyan curves), the response of the sample to the pump stimulation is identical: near *t* = 0 (*t* is the time delay between the pump and the probe), Δ*R*/*R*_0_ undergoes a rapid negative offset due to electrons excitations by a pump energy of 1.6 eV (755 nm), which is higher than the energy gap of 0.6 eV. This rapid photoexcitation is followed by a few picoseconds of relaxation to a nonzero thermalized state. Upon further heating (up to 347 K, Fig. 2a), the relaxation becomes increasingly suppressed, indicating the emergence of charge carriers that are supplied by metallic domains, which evolve with increasing temperature. The latter is also demonstrated by Fig. 1a, where at 340 K, the resistance of VO_2_ drops faster than the exponential function, thus, deviating from the Arrhenius law (marked by the black line) due to the coexistence of the insulating and metallic phases^7^. Next, at 349 K, when the carrier concentration reached its critical value^4^, a sign inversion of the initial Δ*R*/*R*_0_ offset occurs, indicating the overall electronic insulator-to-metal transition (IMT) of VO_2_. Subsequent curves obtained at higher temperatures display an enhanced conductivity of the sample. A similar behavior with temperature for Δ*R*/*R*_0_ is observed for a 250 nm thick VO_2_ film (see Supplementary Information).

Another important feature of the transient reflectivity signal (Fig. 2a) is oscillations with optical phonon frequencies, excited by femtosecond laser pulses. The inset of Fig. 2a shows the extracted coherent oscillations at four different temperatures. Since the phonon modes are the signatures of the atom arrangement in the crystal lattice, their temperature dynamics trace the structural evolution of the sample. In order to reveal the crystallographic transformations of the VO_2_ film in a frequency domain, the transient oscillations were processed by the fast Fourier transformation (FFT). The resulting temperature-dependent frequency map of the coherent phonons in VO_2_ is shown in Fig. 2b and the representative FFT spectra are shown in Fig. 2c–e.

Figure 2b,c show five phonon modes at 4.38 THz (P1), 5.80 THz (P2), 6.07 THz (P3), 6.65 THz (P4) and 6.80 THz (P5) in the vicinity of room temperature (inset of Fig. 2c exhibits the Lorentzian function fitting details). Among these, the P1, P2, and P4 phonons are consistent with those originated from the oscillations of V-atoms in the monoclinic M1 phase of VO_2_ having a ${C}_{2h}^{5}$ space group^13,37,44^, while P3^23,39,45^ and P5^45,46^ are from the monoclinic M2 phase with ${C}_{2h}^{3}$ space group. Note that an observation of the P3 and P5 peaks from the M2 phase already at 303 K is not typical for undoped VO_2_. This can be attributed to a considerably strained state of the film, which is confirmed by high-resolution transmission electron microscopy (HRTEM) imaging of a VO_2_/AlN cross-section, which is analyzed in detail in Supplementary Fig. S4.

Black circles in Fig. 2b mark the precise values of the phonon frequencies, as determined by fitting of the FFT spectra (Fig. 2c–e). Tracing the temperature evolution of the phonon modes, the red shift of the P2-P5 phonons are observed in the vicinity of *T*_IMT_ (in the range of 345–353 K) due to heat-induced lattice expansion and phonon softening^47,48^. Upon further heating (see Fig. 2e), when more and more carriers are generated, the phonon vibrations become less pronounced and, above 359 K, they disappear due to the SPT to the rutile phase. The latter was also confirmed by *in-situ* temperature dependent X-ray diffraction measurements not shown here.

Figure 2c,d show gradual reduction of the P2 and P4 peaks intensity on heating. The peaks are fully symmetrical A_g_ doublet phonons, associated with stretching and tilting of dimerized V-V atoms^13^ in the M1 phase. This damping of the M1 peaks is accompanied by intensifying of the P3 peak from the M2 phase (Fig. 2d, curves at 347–349 K), suggesting the mutual transformation from M1 to M2 and, thus, dominance of the M2 phase at higher temperatures (at 347 K ≤ T < 359 K, Fig. 2c,d). On the other hand, another M2 peak, P5, shows opposite to P3 tendency: its intensity continuously reduces on heating and finally, at 349 K (temperature corresponding to *T*_IMT_), when the P3 peak reaches its maximum intensity, the P5 phonon totally vanishes.

To investigate the evolution of the electronic properties in time we have performed a wavelet analysis using VO_2_ oscillatory data measured at different temperatures and using the Gaussian function

$$f(x)=\frac{1}{w\sqrt{2\pi }}{e}^{-{(x-{x}_{c})}^{2}/(2{w}^{2})}$$

with $$w=1ps$$.

**Figure 3:**
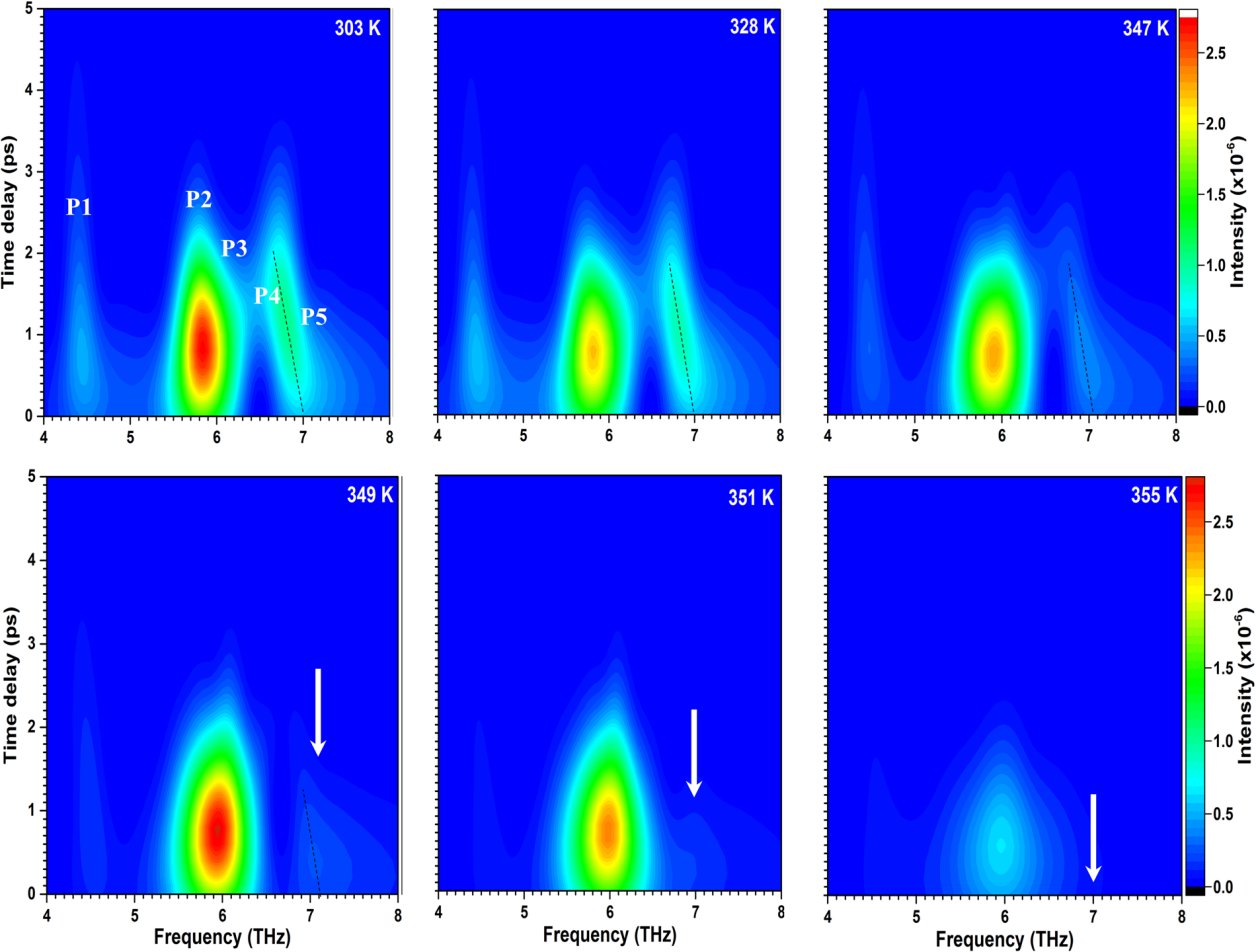
Wavelet transform chronogram of coherent phonons in the VO_2_ film at different temperatures. Black dashed lines show photoinduced softening of the higher frequency phonons, while white arrows mark their temperature-induced damping.

Figure 3 shows the representable frequency-time-intensity maps at different temperatures. It is seen that the phonon frequencies correspond to those obtained by FFT (Fig. 2b–e), but are some broader. Noticeable that, in the range of 303–349 K, the P5 peak from the insulating chain of M2 (IC_M2_) undergoes a significant red shift from ~7.0 to ~6.7 THz with increasing time delay (shown by black dashed line). This behavior can be explained by a rapid pump-induced photoexcitation (please, see the Δ*R*/*R*_0_ offset at time delay ≤200 fs at 303–345 K in Fig. 2a) of the *d*-electrons localized near V-ions composing the IC_M2_, thus, affecting their coherent vibrations. However, in the FFT data (Fig. 2b–e) in the temperature range of 303–349 K, damping of the P5 peak by photoexcitations is not observed, because of insufficient time resolution. It became expressed only above 349 K (on the heat-induced insulator-to-metal transition): the P4 and P5 peaks intensities are strongly reduced due to scattering by charge carriers. On the other hand, at 347–349 K, the charge-density-wave P3 peak from M2 (CDW_M2_) becomes stronger due to M1 → M2 transition. Upon further heating, when more and more carriers are generated, the phonon vibrations become less pronounced and, above 359 K, they disappear (not shown) due to the SPT to the rutile phase. Thus, the results obtained by wavelet analysis are consistent with those, obtained by FFT in the main and supplementary texts (Figs 2 and S2).

A striking discovery is strong damping of the higher frequency P5 phonons from M2 at *T*_IMT_ ≈ 349 K and existence of the lower frequency P3 peak up to 357 K (Fig. 2b,e). These can be explained by structural features of the M2 phase described below.

**Figure 4:**
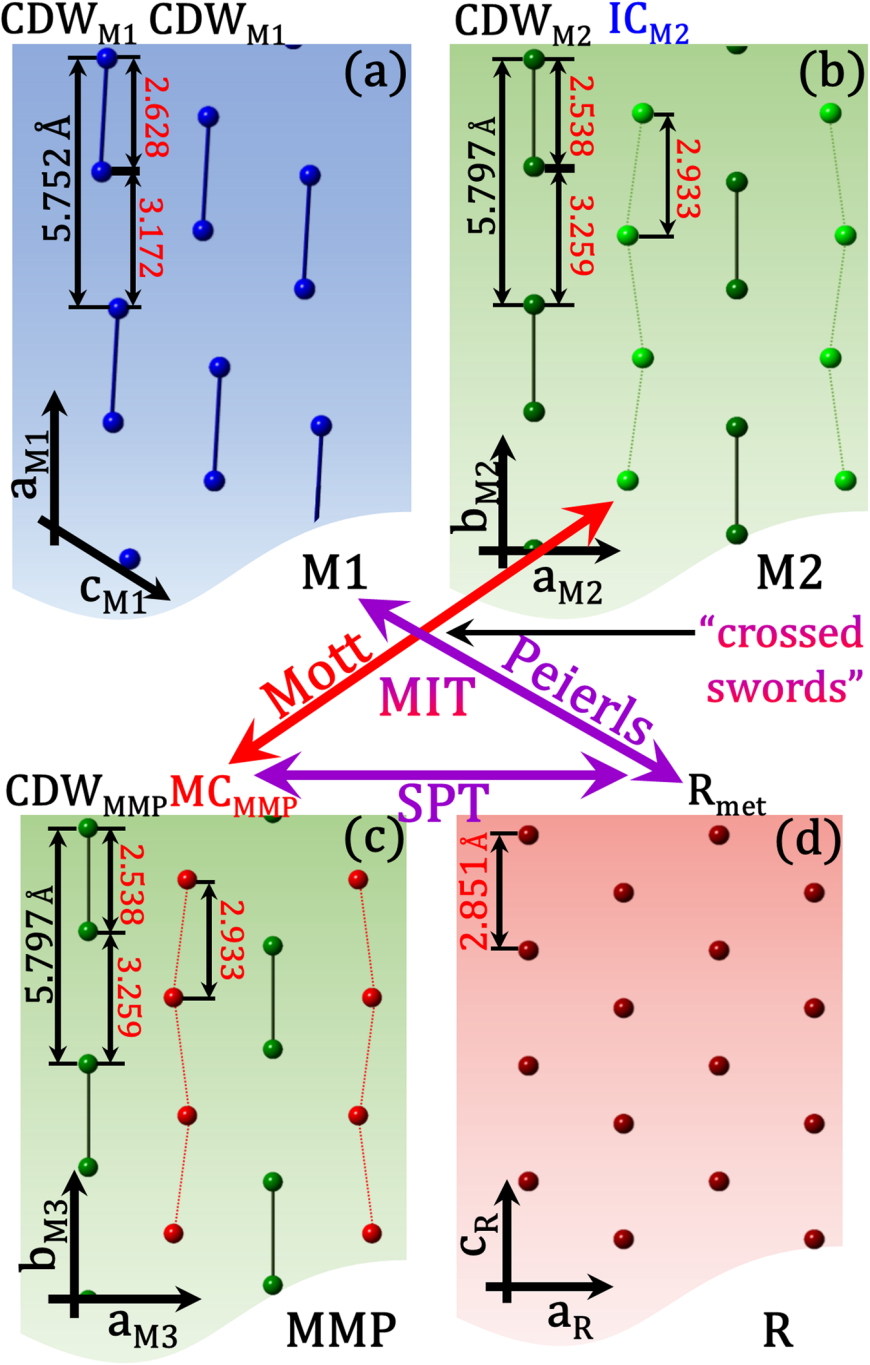
Arrangement of V-atoms in different VO_2_ crystalline structures. (**a**) The monoclinic insulating M1 with commensurate charge-density-wave (CDW_M1_) substructures of metal atoms dimerized along the *a*_M1_ axis. M1 is composed of identical dimer chains of tilted V-atoms with a bond length of 2.628 Å and an interdimer spacing of 3.172 Å. (**b**) The monoclinic insulating M2 with two types of atomic substructures: the commensurate CDW_M2_ with periodically paired atoms and the insulator chain IC_M2_ with equally spaced unpaired V-atoms. (**c**) A monoclinic metallic phase (MMP), which is structurally identical to M2 but with a metallic chain MC_MMP_. (**d**) The rutile metallic structure with identical *R*_met_ substructures of equally spaced V-atoms. Ongoing confrontation or “crossed swords” between Mott (electron-correlation *(e-e)*-driven-IMT in the same structure: M2 ↔ MMP) and Peierls (structurally *(e-ph)*-driven-IMT in different structures: M1 ↔ R) natures of the transition in VO_2_. The Mott IMT (IC → MC) takes place between (**b**) and (**c**). The SPT occurs between (**c**) and (**d**) due to melting of the CDW_MMP_ structure.

Figure 4a shows the schematic of twisted V-V pairs in the M1 phase, which can be considered as a superposition of two lattice distortions of M2^5^. Figure 4b demonstrates the binary V-substructures in the M2 phase. One is the commensurate CDW_M2_ (charge ordering in a distorted lattice) substructure with straightened and alternately positioned metal atoms with spacings of both 2.538 Å between paired and 3.259 Å between unpaired V-V. The other is the insulator-chain (IC_M2_) of a zigzag-like substructure with unpaired V-ions positioned with an equal spacing of 2.933 Å, having strongly correlated localized electrons with the antiferromagnetic half spin. The Mott IMT was predicted to occur through the breakdown of the critical on-site *e-e* Coulomb repulsion in the IC^5,27,29,49^ and its transformation (Fig. 4c) from IC_M2_ to a metallic chain (MC_MMP_)^27,29^. Note that CDW substructure remains same for M2 and MMP (CDW_M2_ ≡ CDW_MMP_). Inset of the Fig. 2d shows the band diagram before IMT for the semiconducting (or insulating) VO_2_ with the IC_M2_ and CDW_M2_ substructures and the band diagram after IMT for the metallic MC_MMP_ and semiconducting (or insulating) CDW_MMP_ substructures.

Based on the above considerations, the two types of observed phonons can be assigned to those coming from the M2 substructures as follows: the P5 phonon, disappearing at *T*_IMT_ as a result of scattering by carriers generated at the IMT, is regarded as that originating from the IC substructure (IC_M2_ in Fig. 4b), while the P3 phonon, able to withstand the scattering effect up to *T*_SPT_ ≈ 359 K because fewer carriers are generated by the corresponding vibrating V-atoms, is interpreted as that coming from the CDW substructure melting on the structural transition to the rutile phase (Fig. 4d). The long-living P3 phonon along with P1 and P2, all persisting up to 359 K, allow to prove that VO_2_ undergoes the Mott insulator-to-metal transition within the monoclinic structure.

## Conclusion

In conclusion, we have studied the temperature dependence of coherent phonons in strained VO_2_ films with monoclinic M2 phase stabilized in a wide temperature range. Observed disappearance of the higher-frequency phonons at *T*_IMT_ is explained by their scattering by charge carriers emerging when the zig-zag insulating chain IC_M2_ substructure of M2, vibration of which creates corresponding phonons, undergoes the Mott transition. While the lower-frequency phonons from the straight charge density wave CDW_M2_ substructure of M2 persist up to *T*_SPT_ (*T*_SPT_ > *T*_IMT_). Thus, we were able the separate two kinds of phonons from M2 and to obtain experimental evidence of the metallic chain (MC) in the monoclinic structure for the first time. This finding proves the Mott transition in VO_2_; it differs from suggestions that the VO_2_ insulator is coupled to the charge order corresponding to the commensurate CDW state and that both *e-e* and *e-ph* interactions are crucial for the phase transition.

## Methods

Epitaxial VO_2_ films on AlN/Si (111) substrates were synthesized using a pulsed laser deposition technique^34^. Additional 100-nm VO_2_ films on Al_2_O_3_ were fabricated in a similar manner. The VO_2_/AlN/Si samples were measured using the femtosecond Ti:Sapphire laser system^39^ with a central wavelength of 755 nm, a pulse duration of 50 fs, and a repetition rate of 560 kHz in reflection geometry. Details on the measurement system are given in the Supplementary Information (Fig. S1). The VO_2_/Al_2_O_3_ samples were measured with transmission geometry using the amplified femtosecond laser system at a pulse width of 45 fs, a repetition rate of 5 kHz, and a pump-probe generation wavelength of 800 nm. Pump fluence was low enough not to trigger the insulator-to-metal transition.

## Electronic supplementary material

Supplementary Information
