# Supplementary material for: Mott transition in chain structure of strained VO2 films revealed by coherent phonons
Source: Sci Rep. 2017 Nov 22;7:16038. doi: 10.1038/s41598-017-16188-6 (PMC5700180; doi:10.1038/s41598-017-16188-6)
Supplement: Supplementary file 1 — Supplementary Information [file 41598_2017_16188_MOESM1_ESM.pdf]

Supplementary Information for

Mott transition in chain structure of strained VO_2 films revealed by coherent phonons

Tetiana V. Slusar[1*], Jin-Cheol Cho[1,2], Hyang-Rok Lee[3], Ji-Wan Kim[4], Seung Jo Yoo[5], Jean-Yves Bigot[4], Ki-Ju Yee[3*] & Hyun-Tak Kim[1,2*]

[1]*Metal-Insulator-Transition Laboratory, Electronics and Telecommunications Research Institute, Daejeon 305-700, Republic of Korea*

[2]*Department of Advanced Device Technology, University of Science and Technology, Daejeon 305-700, Republic of Korea*

[3]*Department of Physics, Chungnam National University, Daejeon 305-764, South Korea*

[4]*Institut de Physique et Chimie des Matériaux de Strasbourg, UMR 7504, CNRS, Université de Strasbourg, 67034 Strasbourg Cedex 02, France*

[5]*Center for Electron Microscopy Research, Korea Basic Science Institute, Daejeon 34133, Republic of Korea*

*Authors to whom correspondence should be addressed. Electronic mail: htkim@etri.re.kr, kyee@cnu.ac.kr, tslusar@etri.re.kr

1. Setup for pump-probe measurements

Figure S1 shows a schematic of the pump-probe spectroscopy setup. The VO_2/AlN/Si samples were measured using the femtosecond Ti:Sapphire laser system [40] with a central wavelength of 755 nm, a pulse duration of 50 fs, and a repetition rate of 560 kHz in reflection geometry (R beams). In the system, the output pulse is divided by the beam splitter BS1 into a pump, exciting the VO_2 film, and a probe, for monitoring the temporal response of the sample for photoexcitations. The intensity distributions are approximately 60 % and 40 % for the pump and probe arms, respectively. The pump beam then enters a shaker, resulting in a time delay of up to ten picoseconds. The consequent mirrors, M2 and M4, send the beam to the VO_2 surface, where it is focused within a spot of 10 μm in diameter, inducing coherent oscillations. The probe beam, with a spot size less than 10 μm (in order to ensure the sensing of only the uniformly excited VO_2 region only), passes BS2, resulting in two beams: one is sent to the balanced detector as a reference signal (R_r), and the other is incident at the sample within the surface area excited by the pump pulse. After being reflected, the pump signal is blocked, while the probe signal, R, carrying the response signal, is directed by M5 to the detector. Thus, the two signals, R_r and R, from the detector are compared, and the raw data for the changes in reflectivity, as shown in Figs. 2*a* & S2*a*, is obtained as follows: $(R - R_r)/R_0 = \Delta R/R_0$, where R_0 is the reflected intensity without the pump beam. The evolution with the time delay provides information regarding the dynamics of the electronic and structural states of VO_2.

The VO_2/Al_2O_3 samples were measured with transmission geometry (T beam in Fig. S1) using the amplified femtosecond laser system at a pulse width of 45 fs, a repetition rate of 5 kHz, and a pump-probe generation wavelength of 800 nm. Pump fluence was set at 1.1 mJ/cm^2, which is low enough not to trigger the insulator-to-metal transition (IMT).

2. Pump-probe measurements of VO_2/AlN/Si

Fig. S2*a* shows the transient reflectivity $\Delta R/R_0$ response, measured at different temperatures, for 250 nm VO_2 film on AlN/Si. The $\Delta R/R_0$ curves are very similar to those, obtained for the 120 nm VO_2 on AlN/Si (Fig. 2*a* of the main text). In low temperature

regions, at 303-323 K (blue and cyan curves), the response of the sample to the pump pulse is identical: near *zero* time delay between the pump and the probe, $\Delta R/R_0$ undergoes a fast negative offset, followed by a few picoseconds of relaxation to a nonzero thermalized state. At higher temperatures (up to 347 K), the relaxation becomes increasingly suppressed by charge carriers generated by nucleating metallic VO_2 domains. In this region, the insulating and metallic phases coexist, which is also supported by deviation of the resistance (above 335 K) from the exponential law (straight black line in Fig. S2*b*), similar to that observed in Fig. 1*a*. In general, both curves for resistance vs. temperature for 120 and 250 nm VO_2 films are very similar, as shown in Fig. S2*b* for comparison. Some differences are seen in the amplitude of the resistance change (bigger jump for 250 nm film) and the sharpness of the transition (slightly sharper for 250 nm film). These can be explained by a smaller substrate-induced strain in the thicker film, resulting in better properties. Upon further heating, at 347-349 K, when the carrier concentration reaches its critical value [4], a sign inversion of the initial $\Delta R/R_0$ offset occurs, indicating the global electronic insulator-to-metal transition (IMT) of VO_2. Subsequent curves obtained at higher temperatures display an enhanced conductivity of the sample.

Fig. S2*c* shows the temperature dependent coherent phonon frequency map of the 250 nm sample obtained in a similar manner as those in Fig. 2*b*. Five modes (P1-P5) at about 4.4, 5.8, 6.1, 6.6 and 6.8 THz are exhibited at 303 K. Following the temperature dependent behavior of the phonons with temperature, it is revealed that the higher frequency P4 and P5 phonons disappear at T_{IMT}, while the lower frequency P1 and P3 vibrations persist up to higher temperature values. Note that early damping of the P2 phonon from the M1 structure, accompanied by an increasing P3 peak intensity, means a faster M1→M2 transition, followed by a further insulating M2 to MMP (monoclinic metallic phase) transition, as described in the main text.

3. Pump-probe measurement of VO_2/Al_2O_3

In order to verify the observed tendency for the strained VO_2 films on AlN/Si, we measured the time-resolved properties of the VO_2 films on a sapphire (Al_2O_3) substrate at different temperatures.

Figure S3*a* shows the temperature dependence of relative transmissivity $\Delta T/T$ with marked points (black crosses), at which further optical measurements have been taken.

Fig. S3*b* shows the transient dynamics of $\Delta T/T$, where, at 295 and 332 K (before T_{IMT}), the $\Delta T/T$ curves demonstrate a rapid negative offset near *zero* time delay, followed by a slower relaxation to a nonzero thermalized state. Next, at 335 K (IMT onset), along with the large negative displacement, the curve experiences small positive values, indicating the "birth" of the metallic phase. At 337 K, in the middle of the IMT, the positive offset of $\Delta T/T$ is dominant, because the film become mostly metallic. Note that the curves obtained in the range of 295-337 K are strongly modulated by coherent vibrations, which are not seen above 337 K, when the structural transition to the rutile phase occurs.

Fig. S3*c* shows the FFT spectra obtained from the coherent oscillations of the $\Delta T/T$ curves in Fig. S3*b*. Here, at room temperature, five peaks (P1-P5) from the M1 (P1, P2 and P4) and M2 (P3 and P5) phases are observed. Upon further heating, the P1 mode disappears, because of its relative weakness and high noise level, while other modes persist up 335 K, however, they are lower in intensity. At 337 K, the P5 peak is fully damped. According to the main text and Figs. 2*b*-*e* & S2*c*, the P5 peak originates from the insulating chain (IC_{M2} in Fig. 4*b*) of the M2 phase, while P3 comes from the charge-density-wave substructure (CDW_{M2} in Fig. 4*b*). Since the Mott transition is believed to occur due to the breakdown of electron-electron correlations in the IC [28], disappearance of the P5 phonon can be explained by its scattering by carriers, generated during the IMT (Fig. 4*c*). Other distinguishable peaks (P2, P3 and P4) indicate that the structure of the film is still monoclinic, thus, supporting the electronic transition within the monoclinic structure.

Generally, the observed phonons from VO_2/Al_2O_3 are consistent with those from VO_2/AlN (Figs. 2*b*-*e* & S2*c*), whereas some deviations in their frequencies and less pronounced peaks from the M2 phase can be explained by the smaller lattice constant mismatch between VO_2 and Al_2O_3 and, thus, the reduced strain in the film compared with VO_2/AlN. These confirm the uniqueness of the VO_2/AlN samples with the strong M2 phase, stabilized up to higher temperatures, providing the ability to reveal the nature of the IMT in VO_2.

4. High-resolution transmission electron microscopy (HRTEM) measurements

As demonstrated above, the presence of the monoclinic structure (in VO_2/AlN/Si) up to atypically high temperatures, as well as the coexistence of the M1 and M2 phases near room temperature (Figs. 2*b-d* and Fig. S2*c*) can be explained by the structural analysis of the VO_2 films measured by HRTEM.

Figure S4*a* shows an HRTEM image with FFT patterns of the VO_2/AlN cross-section. Similar to a previous work [35], the zone axis of the AlN substrate is defined as $[2\bar{1}\bar{1}0]$ (not shown), but there is a prominent distinction in the V-atoms arrangement (see white dashed squares and adjacent FFT patterns). Careful analysis of the interplanar spacing and diffraction spot intensities reveals that these two areas represent two monoclinic phases of VO_2: M1 and M2. Magnified white dashed areas and modeled structures with growth directions of $[010]_{M1}$ ($[100]_{M1}$ zone axis) and $[001]_{M2}$ ($[110]_{M2}$ zone axis) are shown in Figs. S4*b* and *c*.

Figure S4*d* shows the schematic image based on the data extracted from the HRTEM image (Figs. S4*a-c*), depicting the in-plane alignment of VO_2 (sky-blue-colored a_{M1} and c_{M1} axes of the M1 and green-colored a_{M2} and b_{M2} axes of the M2 phases) and AlN (dark-blue-colored tetrahedral unit cells with a_{AlN} lattice constant). Considering the lattice constants of AlN [35], M1-VO_2 [1], and M2-VO_2 [22], the VO_2 film is shown to be under in-plane tensile strains of approximately -7.2 and -13.2 % along a_{M1} and c_{M1}, respectively, whereas it is stretched along a_{M2} by -2.5 % and compressed in the b_{M2} directions by +7.8 %. From the requirements of the minimal lattice constant mismatch at the film-substrate interface, the emergence of the M2 phase at room temperature is preferred. On the other hand, the formation energy of the M2 phase is slightly higher than that of M1, but the difference is blurred with increasing temperature [2, 3], which explains the dominance of the M2 phase near T_{IMT} (P3>P2 in the vicinity of 350 K in Figs. 2*b*,*d*,*e*).

Furthermore, the alignment of the monoclinic VO_2, obtained from HRTEM (Fig. S4*a*), and the expected alignment (from crystallographic considerations) of the rutile (at high temperatures, after the structural phase transition) VO_2 film on AlN substrate, result in the following film-substrate epitaxial relationship (Fig. S4*d*): $(010)_{M1}$+$(001)_{M2}$//$(0001)_{AlN}$ – at low temperatures, before SPT; $(100)_R$//$(0001)_{AlN}$ – at high

temperatures, after SPT. From this, the in-plane area of the VO_2 unit cell for the M1, M2 and R phases, as well as the respective area of the underlying AlN are calculated (see Fig. S4*f*). The relative VO_2/AlN in-plane area mismatch is shown in Fig. S4*e*. The smallest mismatch (-9.3 %, where “-” stands for stretched film by tensile strain from the substrate) is obtained for M2-VO_2 on AlN, the intermediate (-16.0 %) – for R-VO_2, and the largest (-20.1 %) – for M1-VO_2. This explains the advantage of the M2 phase, over M1, for the low-temperature VO_2 film and the further delay of the transition to the rutile phase, enabling the observation of the electronic transition into the monoclinic metal phase with two substructures.

References

1. Longo, J. M., Kierkegaard, P. A Refinement of the Structure of VO_2. *Acta. chem. scand.* **24**, 420-426 (1970).
2. Pouget, J. P., Launois, H., D'Haenens, J. P., Merenda, P., Rice, T. M. Electron Localization Induced by Uniaxial Stress in Pure VO_2. *Phys. Rev. Lett.* **35**, 873-875 (1975).
3. Park, J. H. *et.al.* Measurement of a solid-state triple point at the metal–insulator transition in VO_2. *Nature* **500**, 431-434 (2013).

Supplementary figures

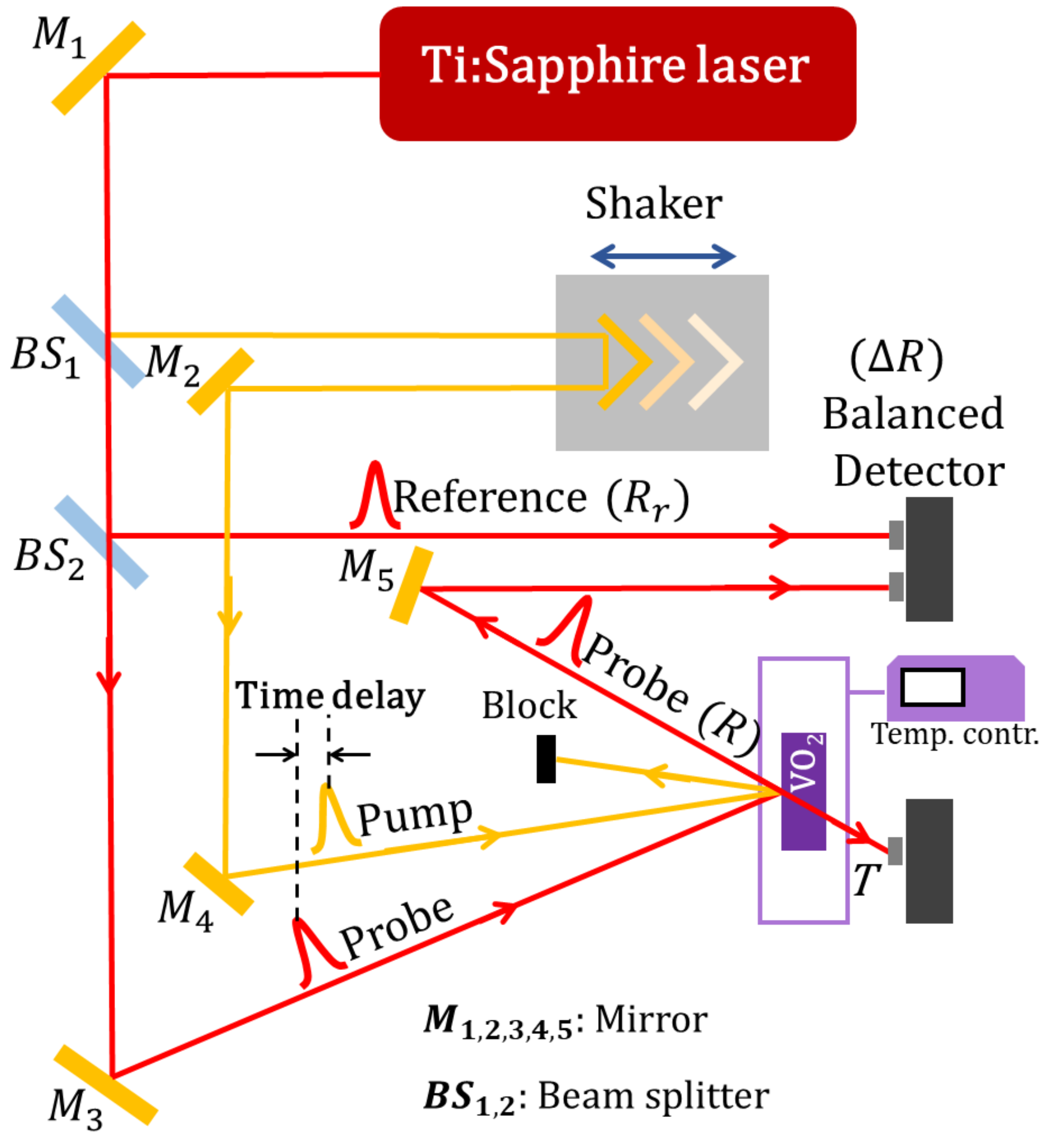

Figure S1. The schematic of the pump-probe setup.

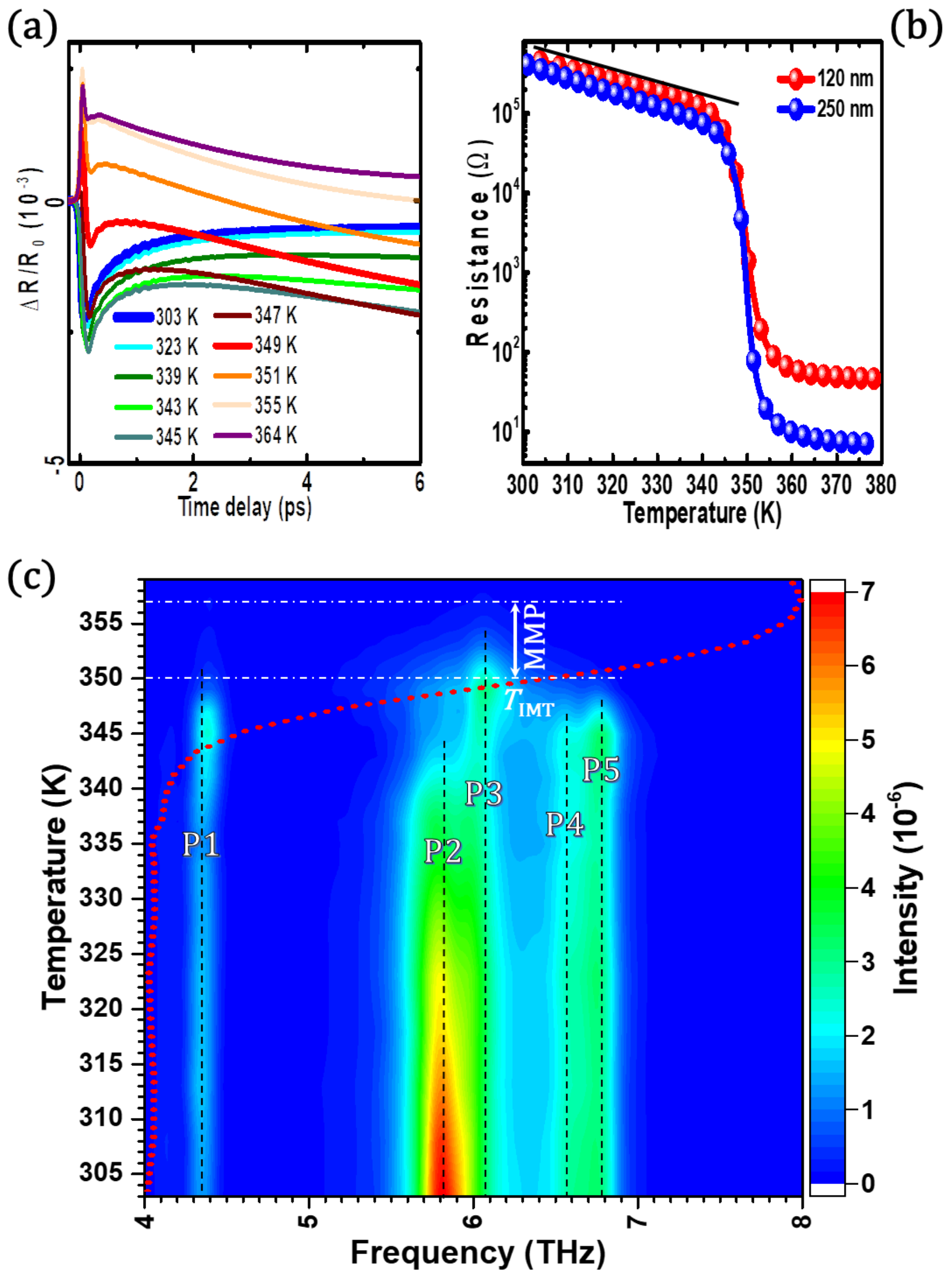

Figure S2. (*a*) Temperature dependence of transient reflectivity for 250 nm VO_2 on AlN/Si. (*b*) Resistance-temperature curves for the 120 and 250 nm VO_2 films on AlN/Si substrate. The black line depicts the Arrhenius law resistance dependency in the insulating state and its deviation from the exponential law, when the metallic puddles emerge and IMT occurs. (*c*) Temperature-frequency map of the five coherent phonons (P1-P5) with the red-dotted curve for normalized probe reflectivity (R/R_0), simultaneously revealing the structural and electronic states of the 250 nm VO_2 film.

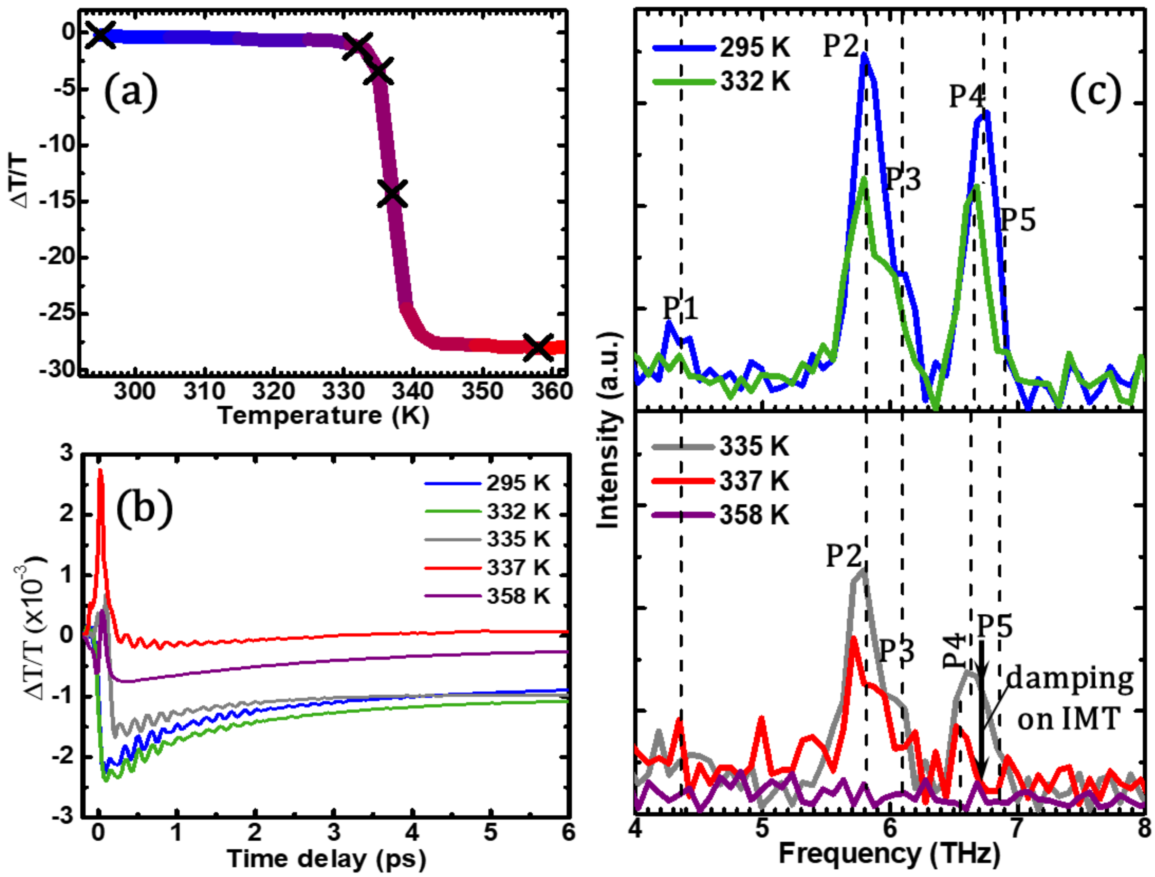

Figure S3. (*a*) Temperature dependence of relative transmissivity Δ*T*/*T* for VO_2/Al_2O_3. (*b*) Temperature dependence of transient Δ*T*/*T* for VO_2/Al_2O_3. (*c*) FFT spectra with five coherent phonon peaks originating from the M1 (P1, P2 and P4) and M2 (P3 and P5) phases.

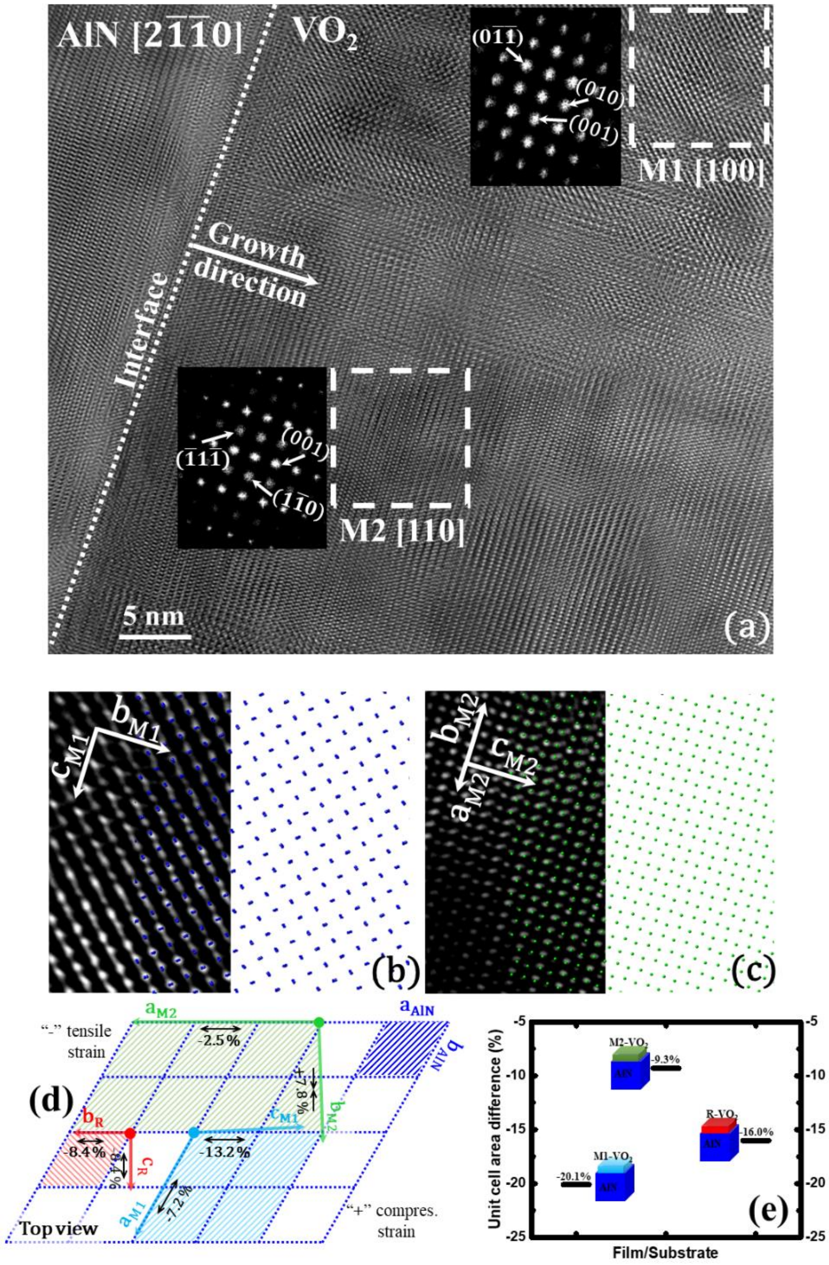

Structure	M1-VO_2	M2-VO_2	R-VO_2	Tetrahedral AlN (f)		
Out-of-plane orientation	*b*-axis	*c*-axis	*a*-axis	*c*-axis		
In-plane area, Å^2	($a_{M1}\times c_{M1}$)	($a_{M2}\times b_{M2}$)	($b_R\times c_R$)	($2a_{AlN}\times 2b_{AlN}$) for M1	($3a_{AlN}\times D_{AlN}$) for M2	($a_{AlN}\times b_{AlN}$) for R
	30.96	52.56	8.13	38.74	57.98	9.68

Figure S4

(*a*) HRTEM image of the VO_2/AlN cross-section with two FFT patterns from the different VO_2 structures M1 and M2 (white dashed squares) observed at room temperature.

(*b*, *c*) Magnified white-dashed area of M1 and M2 taken from (*a*) and modeled structures along $[010]_{M1}$ growth direction and $[100]_{M1}$ zone axis for M1-VO_2 and $[001]_{M2}$ growth direction and $[110]_{M2}$ zone axis for M2-VO_2. (*d*) Top view of the in-plane alignment of the M1 (sky-blue-colored a_{M1} and c_{M1} axes), M2 (green-colored a_{M2} and b_{M2} axes) and R (red-colored b_R and c_R axes) phases of VO_2 with respect to AlN (dark-blue-colored tetrahedral unit cells with a_{AlN} and b_{AlN} lattice constant and D_{AlN} – short diagonal of two AlN unit cells used for calculations in (*f*). Dashed areas mark the underlying AlN surface covered by VO_2 in different phases (M1, M2 and R), used for the film-substrate in-plane area mismatch calculations (*e*, *f*).
